# Overexpression of fibroblast growth factor receptor 2 in bone marrow mesenchymal stem cells enhances osteogenesis and promotes critical cranial bone defect regeneration

**DOI:** 10.3389/fcell.2023.1208239

**Published:** 2023-05-17

**Authors:** Yiwen Zhou, Peixiang Zhu, Siyu Shen, Yanyi Wang, Baochao Li, Baosheng Guo, Huang Li

**Affiliations:** ^1^ Department of Orthodontics, Nanjing Stomatological Hospital, Medical School of Nanjing University, Nanjing, China; ^2^ Medical School of Nanjing University, Nanjing, China

**Keywords:** fibroblast growth factor receptor 2, mesenchymal stem cells, hydrogels, tissue engineering, bone regeneration

## Abstract

**Background:** Reconstruction of cranial bone defects is one of the most challenging problems in reconstructive surgery, and several biological tissue engineering methods have been used to promote bone repair, such as genetic engineering of bone marrow mesenchymal stem cells (BMSCs). Fibroblast growth factor receptor 2 (*Fgfr2*) is an important regulator of bone construction and can be used as a potential gene editing site. However, its role in the osteogenesis process of BMSCs remains unclear. This article clarifies the function of *Fgfr2* in BMSCs and explores the role of *Fgfr2*-overexpressed BMSCs carried by light-induced porous hydrogel (GelMA) in the repair of cranial bone defects.

**Methods:** Lenti-virus was used to overexpress *Fgfr2* in BMSCs, and cell counting kit-8, transwell, and flow cytometry assays were conducted to investigate the proliferation, migration, and characteristics. After 0, 3, 7, and 10 days of osteogenic or chondrogenic induction, the changes in osteogenic and chondrogenic ability were detected by real-time PCR, western blot, alkaline phosphatase staining, alizarin Red staining, and alcian blue staining. To investigate the viability of BMSCs carried by GelMA, calcein and propyl iodide staining were carried out as well. Finally, a critical cranial bone defect model was established in 6-week-old male mice and micro-computerized tomography, masson staining, and immunohistochemistry of OCN were conducted to test the bone regeneration properties of implanting *Fgfr2*-overexpressed BMSCs with GelMA in cranial bone defects over 6 weeks.

**Results:** Overexpression of *Fgfr2* in BMSCs significantly promoted cell proliferation and migration and increased the percentage of CD200+CD105+ cells. After osteogenic and chondrogenic induction, *Fgfr2* overexpression enhanced both osteogenic and chondrogenic ability. Furthermore, in cranial bone defect regeneration, BMSCs carried by light-induced GelMA showed favorable biocompatibility, and *Fgfr2*-overexpressed BMSCs induced superior cranial bone regeneration compared to a normal BMSCs group and an untreated blank group.

**Conclusion:** *In vitro*, *Fgfr2* enhanced the proliferation, migration, and stemness of BMSCs and promoted osteogenesis and chondrogenesis after parallel induction. *In vivo*, BMSCs with *Fgfr2* overexpression carried by GelMA showed favorable performance in treating critical cranial bone defects. This study clarifies the multiple functions of Fgfr2 in BMSCs and provides a new method for future tissue engineering.

## 1 Introduction

Cranial bone defects are some of the most common surgical injuries and can be caused by accidental trauma, severe infection, or tumor excision (Xie et al., 2021). Delayed or non-healing of bone defects often occurs in some cases, such as severe wound infections, extensive bone defects, and diabetes, have generated a high demand for more effective treatments (Quan et al., 2021). Bone marrow mesenchymal stem cells (BMSCs) with rapid self-renewal and multidirectional differentiation potential, are considered to be a new direction for bone defect repair and regeneration (Jin and Lee, 2018; Krampera and Le Blanc, 2021). However, several challenges remain in maintaining optimal cell potency and viability during the expansion, storage, and eventual implantation of the BMSCs and BMSCs tend to senesce and lose multidifferentiation ability with time in culture (Derubeis and Cancedda, 2004; Zhou et al., 2021). To overcome this limitation and enhance the effect of BMSCs in bone regeneration, a variety of biological materials have been developed as carriers of BMSCs to fill in bone defect areas and promote bone formation (Montoya et al., 2021). Meanwhile, tissue engineering methods for BMSCs (such as gene editing technology) that can achieve stronger osteogenic repair ability have been highly anticipated.

Fibroblast growth factor receptor 2 (*Fgfr*2) is a cell membrane surface receptor that plays a critical role in bone development and homeostasis (Marie, 2012; Coffin et al., 2018; Coll et al., 2019). *Fgfr*2 is expressed during embryonic bone development and is involved in the osteogenesis of both skull and long bones (Su et al., 2014). Abnormal mutations in *Fgfr2* can affect both intramembranous ossification and entochondrostosis, leading to dysplasia of skeletal morphology and structure (Wilkie et al., 1995; Merrill et al., 2012; Sargar et al., 2017). It has also been found to be highly expressed during fracture healing, especially during the osteogenesis stage (Schmid et al., 2009), which indicates the potential for *Fgfr2* to be used as a gene editing site in bone regeneration. However, both the exact function of *Fgfr2* in mesenchymal stem cells and whether *Fgfr*2 can be used as an engineering site to promote bone repair remain unclear.

Hydrogels, a range of natural and synthetic materials and biopolymers, have been used in bone regeneration for several years (Filippi et al., 2020). By simulating the extracellular matrix microenvironment and providing adequate water, hydrogels have good biocompatibility, allowing cell proliferation and multidirectional induced differentiation. Simultaneously, it has perfect diversity in geometry and can be used as injectable for any defect area implantation (Lim et al., 2019).

In this study, we used Lenti-virus to overexpress *Fgfr2* in BMSCs to explore the effects of excessive *Fgfr2* on proliferation, migration, anti-apoptosis ability and cell stemness. Subsequently, we applied both osteogenic and chondrogenic induction to observe whether *Fgfr2* overexpression could promote osteogenic differentiation and chondrogenic differentiation of BMSCs. Finally, BMSCs with *Fgfr2* overexpression were mixed with light-induced porous gelatin methacryloyl hydrogel (GelMA) and implanted into mouse cranial critical bone defects to detect its therapeutic effects. Our study thus may elucidate the different roles of *Fgfr2* in mesenchymal stem cells and provide new insight into stem-cell-directed treatment of cranial bone defects.

## 2 Materials and methods

### 2.1 Cell culture

The mice primary BMSCs were purchased from the Cyagen Biosciences Co. (MUBMX-01001). At stem cell state, the BMSCs were cultured with αMEM with 10%FBS and 1% penicillin-streptomycin antibiotics. As for osteogenic induction, 0.1uM dexamethasone (ST1254, Beyotime Biotechnology), 50ug/ml vitamin C (ST1434, Beyotime Biotechnology) and 10 mM *β*-glycerophosphate (G9140-5, Solaribio) were contained in αMEM complete medium. As for chondrogenic induction, 1% ITS (C0341, Beyotime Biotechnology), 0.1uM dexamethasone (ST1254, Beyotime Biotechnology), 37.5ug/ml vitamin C (ST1434, Beyotime Biotechnology), 1 mmol/L sodium pyruvate (ST1661, Beyotime Biotechnology) and 10 ng/ml TGF-β1 (PRP1017, Abbkine Scientific Co.) were contained in high glucose DMEM complete medium. The cells were cultured in the cell incubator (37 °C. 5% CO_2_) and changed medium every 2 days.

### 2.2 Lenti-virus transfection

The Lenti-virus was constructed by Syngen Technology. With the *Fgfr2* overexpression Lenti-virus (Lenti-*Fgfr2*), a control virus was also constructed (Lenti-control). Both Lenti-*Fgfr2* and Lenti-control were transfected into BMSCs and the cells were subsequently named as the *Fgfr2* overexpression group and the Con group. After screening the optimum multiplicity of infection (MOI), we chose MOI = 50 for the transfection. The virus supernatant was used with 10ug/ml Polybrene (C0351, Beyotime Biotechnology) to transfect BMSCs for 8 h. After 48 h, the total RNA and protein were extracted to test the transfection efficiency.

### 2.3 Total RNA extraction and real-time qPCR

The total RNA of BMSCs was extracted by RNAeasy™ Animal RNA Extraction Kit (R0026, Beyotime Biotechnology) according to the manufactures recommended procedure. After determining the concentration, reverse transcription was performed using the Evo M-MLV RT Kit (AG11728, Accurate Biology). The real-time qPCR process was done on the ABI ViiA 7(Life Technologies) with the SYBR Green Pro Taq HS Kit (AG11701, Accurate Biology) following the recommended protocol.

### 2.4 Total proteins extraction and Western blot

Total proteins of BMSCs were extracted using a total protein extraction buffer (P0013, Beyotime Biotechnology). BCA protein quantification kit (P0010S, Beyotime Biotechnology) was used to quantify the protein contents. The obtained protein was denatured with SDS loading buffer (D0071, Beyotime Biotechnology). Protein was fractionated by SDS polyacrylamide gel electrophoresis and incubated overnight at 4 °C with antibodies against FGFR2 (1:2000, ab10648, Abcam), RUNX2 (1:1000, ab236639, Abcam), COLII (1:1000, 28459-1-AP, Proteintech) and GAPDH (1:50000, AC033, Abclonal), respectively. After washing 3 times with TBST, the membranes were incubated with a secondary antibody. Finally, the proteins were detected by ECL luminous fluid (P0018S, Beyotime Biotechnology).

### 2.5 CCK-8 assay

The CCK-8 assay was carried out followed by the protocol provided by the manufacturer (C0037, Beyotime Biotechnology). In brief, 1000 cells were plated in 96-well plates for cell proliferation detection. At 0 h, 24 h, 48 h, and 72 h, the CCK-8 kit was added and plates were cultured in the cell incubator for 2 h. The absorbance was detected with a microplate reader at 405 nm wavelength.

### 2.6 Transwell assay

The vertical migration ability of BMSCs was performed by a transwell system (14,141, 8um, Polycabonate membrane, Labselect). BMSCs were seeded at a density of 1 × 10^5^ per well. After 24 h, collected the chamber and removed the cells on the upper side of the Transwell filter. The cells on the downside of the filter were stained with crystal violet (C0121, Beyotime Biotechnology) and photographed. Later, the cells were incubated with 200ul decoloring solution (33% glacial acetic acid) and detected the quantification of transwell results with a microplate reader at 570 nm wavelength.

### 2.7 Scratch assay

The scratch healing experiment was performed using the scratch plug-in method. The scratch wound was made with a fixed scratch 500 μm wide. Suspensions were prepared with cells at a density of 3 × 10^5^/mL, and a 70 μl cell suspension was seeded into each plug-in. When the degree of fusion of the cells reached 85%, the plug-ins were removed. Cells were washed with PBS and cultured in αMEM complete medium. The width of the scratch was observed and imaged under a microscope (0h, 24h, 48h, 72 h).

### 2.8 Flow cytometry

For the CD200 and CD105 staining, the cells were digested and stained by the CD200 (12-5200-82, ThermoFisher Scientific) and CD105 (17-1051-82,ThermoFisher Scientific) antibodies for 30min. After 2 times washing, the mean fluorescence intensities of CD200 and CD105 were detected by BD FACS Aria and the results were analyzed by FlowJo 10.5.3.

### 2.9 Tunel staining

The tunel staining was applied to BMSCs after 72 h serum shock following the instruction of Tunel staining kit (A112-02, Vazyme). After staining, images were taken under a confocal microscopy and the percentage of Tunel positive cells was counted by Photoshop.

### 2.10 ALP staining

After osteogenic induction for 7 days, BMSCs were fixed with 4% PFA and incubated with BCIP/NBT Alkaline phosphatase color developing kit (C3206, Beyotime Biotechnology) for 30 min. After washing by PBS, the staining cells were photographed by a stereoscope and a microscope.

### 2.11 Alizarin Red staining

After osteogenic induction for 14 days, BMSCs were fixed with 4% PFA and incubated with 0.2% Alizarin Red S Solution (G1450, Solarbio) for 30 min. After washing with PBS, the staining cells were photographed by a stereoscope and a microscope.

### 2.12 Alcian blue staining

After chondrogenic induction for 14 days, BMSCs were fixed with 4% PFA and incubated with an Alcian blue Staining Kit (C0153S, Beyotime Biotechnology) for 1 h. After washing with PBS, the staining cells were photographed by a stereoscope and a microscope.

### 2.13 Light-induced porous gelatin methacryloyl hydrogel (GelMA)

The light-induced GelMA was purchased from Engineering For Life Co. (5%, EFL-DM-60). BMSCs were mixed with the GelMA after it was configured and filtered according to the instructions. The mixed GelMA was then cured under 405 nm blue light (EFL-LS-1600-405). For *in vitro* experiments, the density of BMSCs was 5 × 10^4^/ml, and for *in vivo* experiments, the density of BMSCs was 1 × 10^7^/ml.

### 2.14 Live and dead staining

The live and dead assay was carried out using a Calcein/PI cell viability assay kit (C2015S, Beyotime Biotechnology). GelMA mixed with BMSCs in each well was added with 1 ml Calcein AM/PI solution, and incubated for 1 h at 37°C avoided light. Then, the live/dead cells were observed under a fluorescence microscope and a confocal microscope.

### 2.15 Mice

**FIGURE 1:**
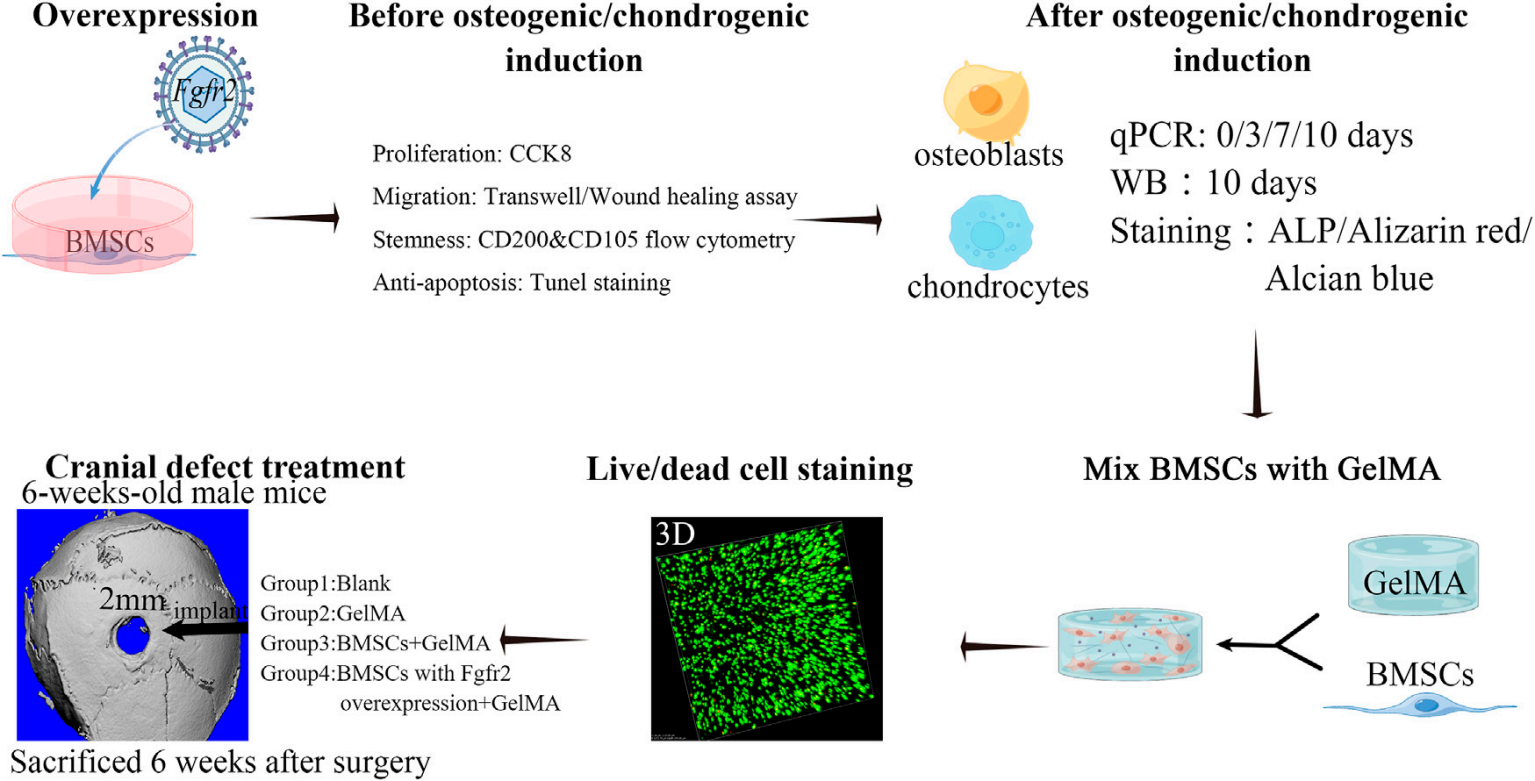
Scheme of experimental design. *Fgfr2* overexpression was performed on BMSCs by Lenti-virus and the proliferation, migration, cell stemness and anti-apoptosis ability were detected. Subsequently, osteogenic induction and chondrogenic induction were added to detect changes in osteogenic differentiation, mineralization and chondrogenic differentiation ability. Furthermore, BMSCs and GelMA were mixed and biocompatibility was confirmed *in vitro*. Then, BMSCs mixed with GelMA were implanted into the cranial bone defect area of mice to observe the regeneration ability.

The 6-week-male C57BL/6 mice (21–23 g) were from Shanghai Bikai Keyi Biotechnology Co. (Certificate No. 20180006044831). A total of 24 mice were used (Figure 1). The mice were anesthetized by gas anesthesia using isoflurane. A round defect of 2 mm in diameter on the parietal bone was made by a trephine drill and then the GelMA with or without BMSCs was implanted into the defect. A blank group was also made as a control. After 6 weeks of repairation, the mice were sacrificed by cervical dislocation following anesthesia. All animal experiments were approved by the Animal Ethics Committee of Nanjing University (Permit No. IACUC——D2303101).

### 2.16 Micro-CT analysis

The skulls of mice in each group were fixed in 4% PFA and scanned by SCANCO vivaCT 80 (MedicalAG). The X-ray voltage was 57 kV and the current was 184μA, with a resolution of 10 μm per pixel. The bone volume/tissue volume (BV/TV) of the defected area was calculated and the 3D images of the defect area were also reconstructed. The Photoshop software was used to measure the area of the bone defect to obtain quantitative data.

### 2.17 Histological staining

The decalcified skulls were dehydrated and then embedded in paraffin. The skulls were then cut into sections for H&E and Masson Staining. For immunofluorescence staining, the sections were incubated with anti-OCN (1:200, A6205, Abclonal) and then incubated with secondary antibodies for inmmunohistochemistry. Slices were sealed with neutral gum and photographed under a microscope.

### 2.18 Statistic analysis

Quantitative data were presented as the mean ± standard error. The statistics were analyzed with an independent *t*-test (two-tailed) by SPSS 15.0. *p < 0.05, **p < 0.01, **p < 0.001.

## 3 Results

### 3.1 Identification of *Fgfr2* overexpression in BSMCs by Lenti-virus

**FIGURE 2:**
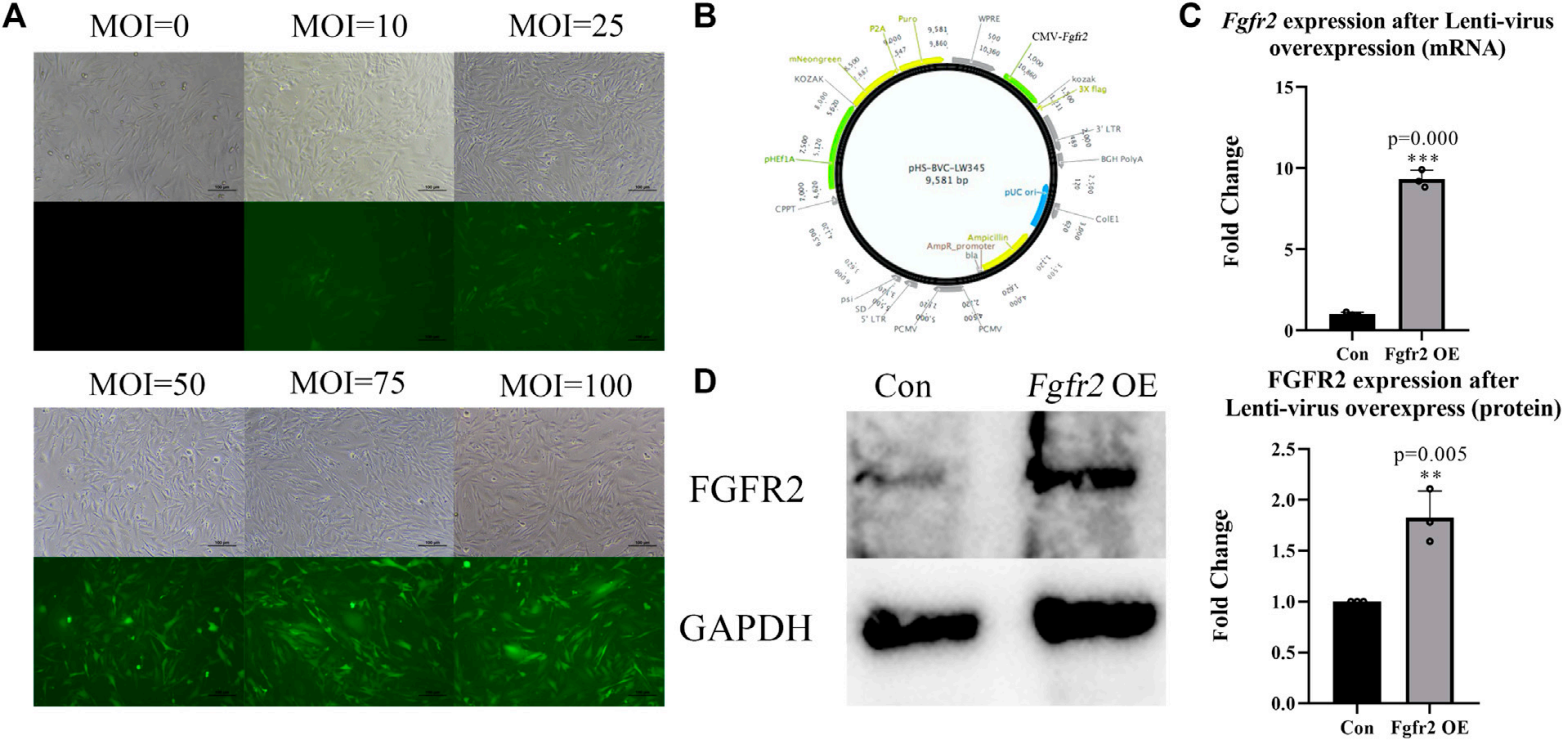
Overexpression of *Fgfr2* in BMSCs. **(A)** After transfecting BMSCs with different MOIs, cellular morphology and fluorescence expression were observed under an inverted microscope and fluorescence microscope, respectively. **(B)** Lenti-virus construction. **(C)** Quantification of *Fgfr2* expression after transfection in RNA level (n = 3). **(D)** The expression of FGFR2 in BMSCs after transfection and quantification (n = 3). The graphs show the mean value ±SD. *p < 0.05, **p < 0.01, ***p < 0.001.

We overexpressed *Fgfr2* in BMSCs by Lenti-virus carrying a green fluorescent protein sequence so that the cells that were successfully transfected would show green fluorescence. First, we screened the optimum multiplicity of infection (MOI) of transfection *in vitro* (Figure 2B) and found that with an increase in MOI, the number of fluorescent cells and fluorescence intensity improved significantly. Considering the potential risk for cells survival condition with high MOI, we selected MOI = 50 as the transfection concentration for BMSCs (the transfection efficiency achieved at 80%).

To identify overexpression efficiency of *Fgfr2*, RNA and protein were also extracted (Figures 2C,D). The results showed that after Lenti-*Fgfr2* transfection, the RNA level of *Fgfr2* was significantly up-regulated (9.31-fold higher, *p* = 0.000), and the protein level was also raised (1.83-fold, *p* = 0.005), suggesting that the overexpression of *Fgfr2* by Lenti-virus was indeed effective.

### 3.2 Overexpression of *Fgfr2* promoted the proliferation, migration, anti-apoptosis ability and cell stemness of BMSCs under a non-induction environment

**FIGURE 3:**
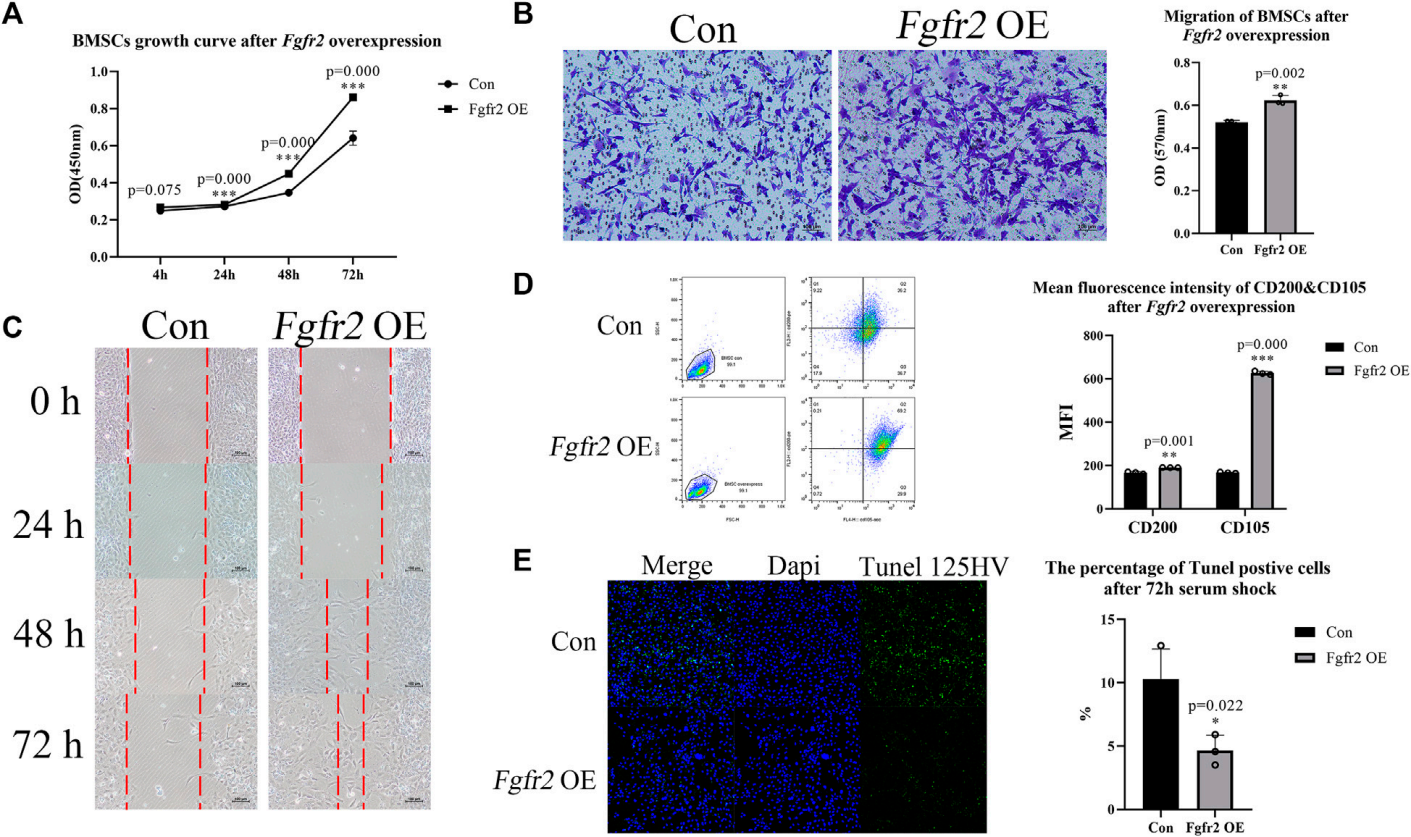
Proliferation, migration, and cell stemness of BMSCs with *Fgfr2* overexpression. **(A)** The proliferation curves of BMSCs after *Fgfr2* overexpression (n = 6). **(B)** The transwell migration assay and quantification of BMSCs after *Fgfr2* overexpression (n = 3). **(C)** Scratch experiment images of BMSCs after *Fgfr2* overexpression (n = 3). Dashed lines represent the scratch width. **(D)** The flow cytometric graphs and quantification of the mean fluorescence intensity (MFI) of CD200 and CD105 after *Fgfr2* overexpression (n = 3). **(E)** Tunel staining and quantification of BMSCs after 72 h serum shock. Graphs show the mean value ±SD. *p < 0.05, **p < 0.01, ***p < 0.001.

In order to understand the effect of *Fgfr2* on the function of BMSCs itself, we first detected the influence of *Fgfr2* overexpression in cell proliferation, migration, and surface markers. CCK8 assay (Figure 3A) showed that the *Fgfr2* overexpression group had a faster proliferation rate compared to the control group at 24 h (1.04-fold, *p* = 0.000), and the raise was more significant at 48 h and 72 h (1.29-fold and 1.34-fold respectively, *p* = 0.000). To test cell migration ability, transwell and scratch assays were both performed as well (Figures 3B,C). These results showed that more BMSCs went across the transwell membranes in the *Fgfr2* overexpression group (1.20-fold in transwell, *p* = 0.002) compared to the control group, and the scratch width was also narrower, indicating the stronger migration ability of BMSCs after *Fgfr2* overexpression. In addition to its effect on proliferation and migration, *Fgfr2* overexpression also enhanced the resistance of BMSCs to apoptosis. 72 h after serum shock, the percentage of tunel positive cells in *Fgfr2*-overexpressing BMSCs was significantly fewer compared with the control group (Figure 3E).

We further performed flow cytometry of CD105 and CD200 to test the changes in surface markers after *Fgfr2* overexpression (Figure 3D). CD105 is a positive marker that is commonly used for BMSC identification, and CD200 can indicate colony-forming ability and osteogenic potential (Lin et al., 2013; Pontikoglou et al., 2016). We observed that the mean fluorescence intensities of CD105 and CD200 were significantly increased after *Fgfr2* overexpression, especially CD105 (3.75-fold, *p* = 0.000). Considering this enhanced proliferation, migration and anti-apoptosis ability, we hypothesize that *Fgfr2* overexpression also promoted the cell stemness of the BMSCs.

### 3.3 Overexpression of *Fgfr2* promoted the osteogenic differentiation and mineralization of BMSCs

**FIGURE 4:**
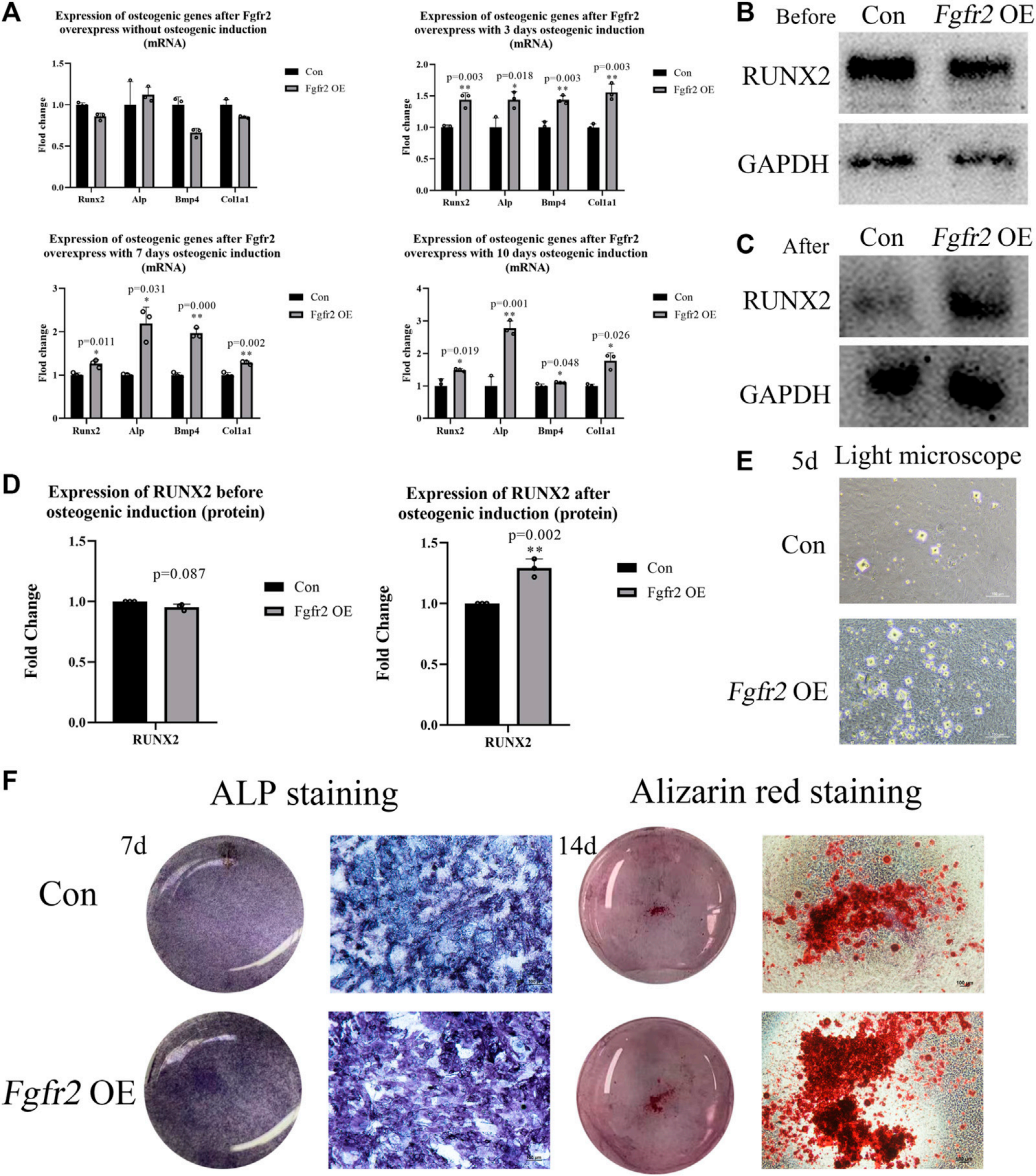
The osteogenic ability of BMSCs with *Fgfr2* overexpression. **(A)** Expression of *Runx2*, *Alp*, *Bmp4*, and *Col1a1* in RNA levels at day 0, 3, 7, and 10 after osteogenic induction (n = 3). **(B–D)** Western blot analysis and quantification of RUNX2 at day 0 and 10 after osteogenic induction (n = 3). **(E)** The representative images of mineralized nodules under a light microscope after 5 days of osteogenic induction. **(F)** The macrograph and micrograph of ALP (10x) and alizarin red staining (4x) of BMSCs after 7 and 14 days of osteogenic induction (n = 3). Graphs show the mean value ±SD. *p < 0.05, **p < 0.01, ***p < 0.001.

*Fgfr2* has been reported to be highly expressed during the differentiation of BMSCs into osteoblasts (Su et al., 2014). Therefore, we investigated the osteogenic differentiation ability of BMSCs after *Fgfr2* overexpression. BMSCs in both the control group and the *Fgfr2* overexpression group were subjected to osteogenic induction for 0, 3, 7, and 10 days, and their RNA was then extracted for detection (Figure 4A). The results showed that compared to the control group, the osteogenic-related genes such as *Runx2*, *Alp*, *Bmp4*, and *Col1a1* were obviously raised after osteogenic induction in the *Fgfr2* overexpression group. On day 3, the up-regulation levels of *Runx2*, *Alp*, *Bmp4*, and *Col1a1* were similar (1.43 to 1.55-fold), and at day 7, the up-regulation levels of *Alp* (2.19-fold, *p* = 0.031) and *Bmp4* (1.97-fold, *p* = 0.000) were the most obviously. When it came to day 10, the up-regulation levels of *Alp* (2.78-fold, *p* = 0.001) and *Col1a1* (1.78-fold, *p* = 0.026) were the highest.

After detecting the osteogenic potential in RNA levels, we also extracted proteins from the BMSCs at day 0 and day 10 (Figures 4B-D). Consistent with the above, RUNX2 expression was not up-regulated without osteogenic induction, but significantly increased at day 10 in the *Fgfr2* overexpression group after osteogenic induction (1.29-fold, *p* = 0.002). These results suggest that *Fgfr2* is a potential regulator of osteogenesis in BMSCs. Thus, we find that although overexpression of *Fgfr2* can enhance the osteogenic potential, it still requires environmental stimulation to drive this process.

To examine the mineralization ability of BMSCs further, ALP and alizarin red stainings were performed. Obvious mineralized nodules could be easily observed under a light microscope in the *Fgfr2* overexpression group at day 5 with osteogenic induction, but the control group only had small mineralized nodules (Figure 4E). Compared to the control group, the *Fgfr2* overexpression group all showed better alkaline phosphatase activity (Figure 4F) and more mineralized nodules with alizarin red staining, demonstrating the superior osteogenic potential of BMSCs after *Fgfr2* overexpression. Therefore, we find that overexpression of *Fgfr2* can significantly promote osteogenic differentiation and mineralization in BMSCs.

### 3.4 Overexpression of *Fgfr2* promoted the chondrogenic differentiation of BMSCs

**FIGURE 5:**
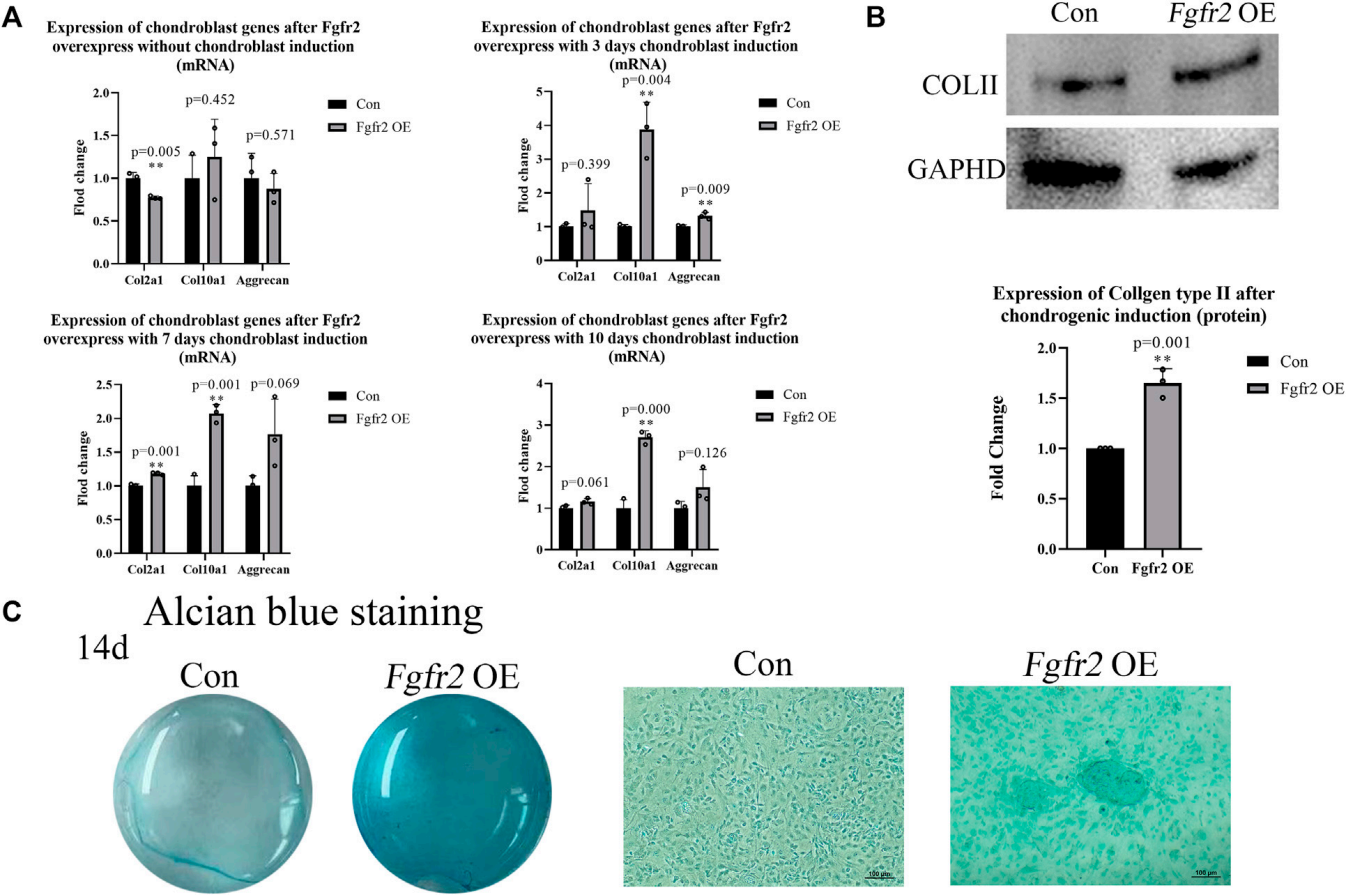
Chondrogenic differentiation potential of BMSCs after *Fgfr2* overexpression. **(A)** Chondrogenic marker RNA expression at 0, 3, 7 and 10 days of induction (n = 3). **(B)** Western blot analysis and quantification of COLII in BMSCs at 0 and 10 days of induction (n = 3). **(C)** The macrograph and micrograph (4x) of Alcian blue staining of BMSCs after chondrogenic induction for 14 days (n = 3). Graphs show the mean value ±SD.*p < 0.05, **p < 0.01, ***p < 0.001.

*Fgfr2* is involved not only in intramembrane osteogenesis but also in endochondral osteogenesis. In diseases resulting from abnormal *Fgfr2* mutations, such as Apert syndrome (*Fgfr2* P253R mutation), an obvious delay in the intracoronal ossification center has been observed that affects the structure of the skull base and long bones (Yin et al., 2008). Therefore, we speculated that *Fgfr2* overexpression might also promote the differentiation of BMSCs into chondrocytes. To test this, we applied a chondrogenic induction environment to the BMSCs and extracted RNA at day 0, day 3, day 7, and day 10 for chondrogenic marker detection (Figure 5A). The results showed that *Col2a1*, *Col10a1*, and *Aggrecan* were all up-regulated with chondrogenic induction and that *Col10a1* was the most dramatically up-regulated gene (3.88-fold, *p* = 0.004, day 3; 2.07-fold, *p* = 0.001, day 7; 2.70-fold, *p* = 0.000, day 10). Subsequently, the protein of BMSCs at 0 and 10 days after chondrogenic induction was extracted, and the expression of COLII was detected (Figure 5C). Consistent with the RNA results, when compared to the control group, COLII showed significant up-regulation in the *Fgfr2* overexpression group 10 days after induction (1.65-fold, *p* = 0.001).

Alcian blue staining further demonstrated the effect of *Fgfr2* overexpression on the chondrogenic ability of BMSCs (Figure 5B). 14 days after chondrogenic induction, the control group did not show the formation of chondrogenic pellets, but the *Fgfr2* overexpression group had a scattered distribution of chondrogenic pellets, and the cells were more deeply stained with Alcian blue. These results indicated that *Fgfr2* not only participated in the osteogenic differentiation of BMSCs but also promoted chondrogenic differentiation. We find that *Fgfr2* is therefore important for both intramembrane osteogenesis and endochondral osteogenesis in BMSCs.

### 3.5 The biocompatibility of BMSCs carried by light-induced GelMA

**FIGURE 6:**
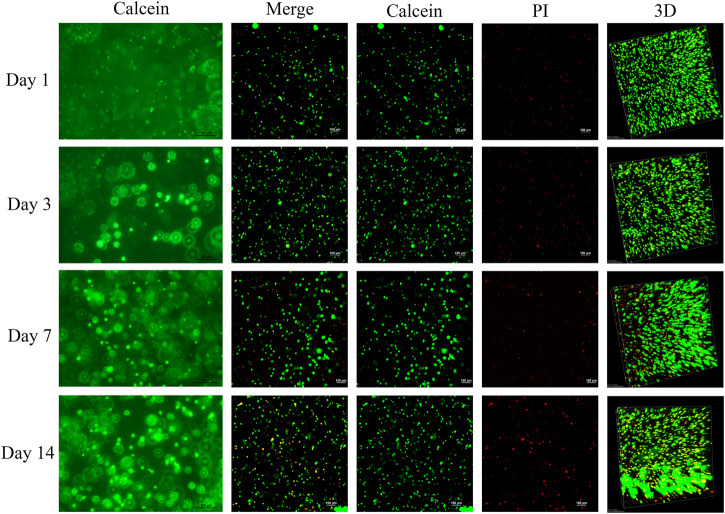
Live/dead cell staining of BMSCs embedded in light-induced GelMA. Images were taken with a fluorescence microscope (first column) and a confocal microscope (second to fifth column). Live cells were stained green by calcein, and dead cells were stained red by PI (*n* = 3).

After demonstrating that *Fgfr2* overexpression could significantly promote the proliferation and osteogenic and chondrogenic differentiation of BMSCs, we wondered whether *Fgfr2* could be used as a novel gene editing site for the treatment of critical cranial bone defects. To investigate this, we selected light-induced porous gelatin methacryloyl hydrogel (5% GelMA) as a scaffold for the BMSCs since it has been shown to be a good three-dimensional scaffold material for cell cultures by supporting the proliferation and multi-lineage differentiation of mesenchymal stem cells (Bashir et al., 2023). We first reconfirmed *in vitro* whether the BMSCs could survive in this GelMA material by calcein/PI staining assay and found that the BMSCs survived quite well in GelMA and proliferated steadily over time (Figure 6). Additionally, many BMSCs began to spread around and connect with each other in the GelMA, suggesting that the BMSCs had begun to form a natural reticular structure. These results indicated that light-induced GelMA did indeed have good biocompatibility with the BMSCs.

### 3.6 BMSCs with *Fgfr2* overexpression carried by light-induced GelMA promoted critical cranial bone defect regeneration

**FIGURE 7:**
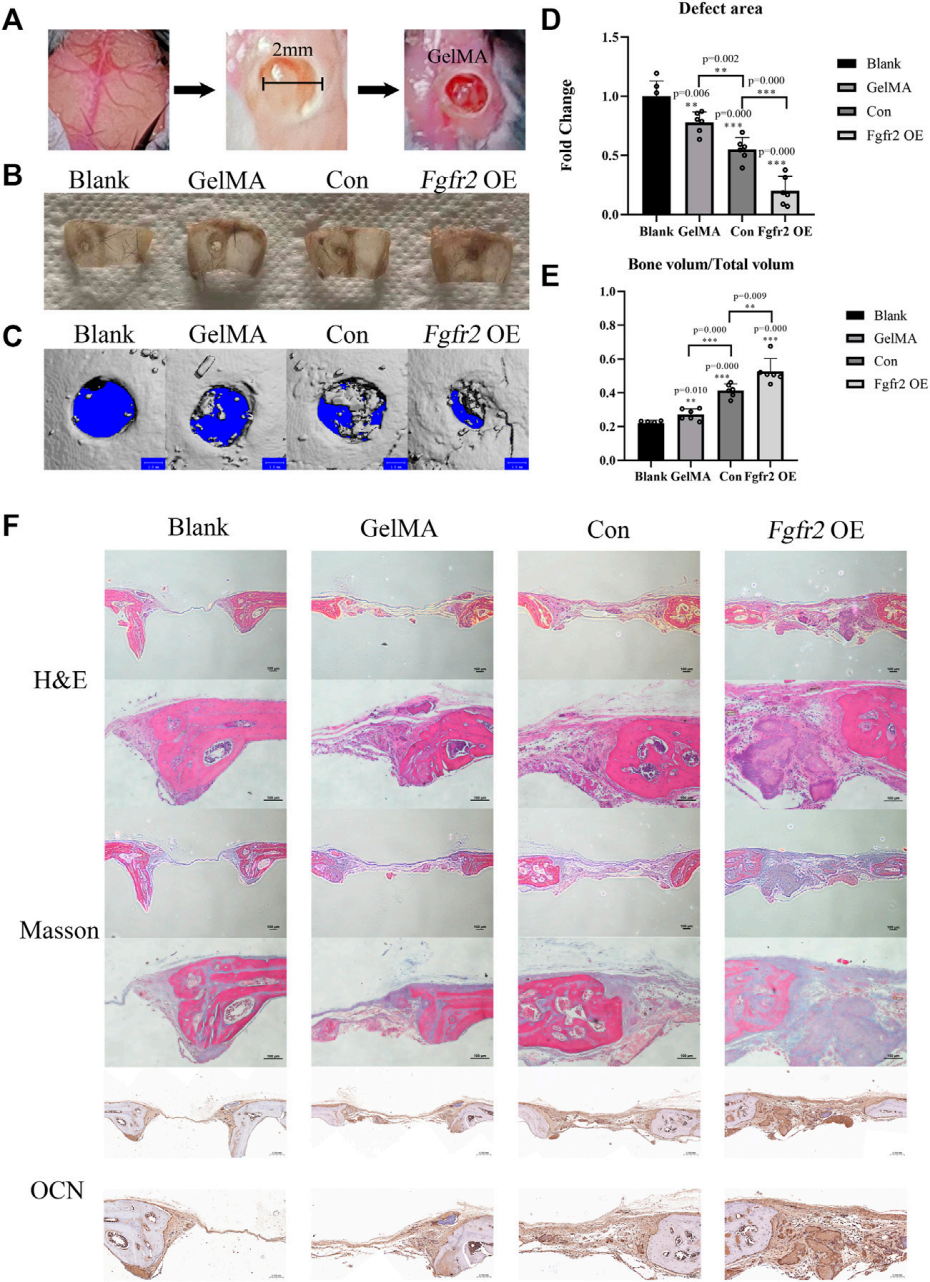
The therapeutic effects of the *Fgfr2* overexpression BMSCs carried by GelMA on mouse cranial bone defects. **(A)** Diagram of skull bone defect modeling and GelMA implantation with 405 nm light induction. **(B)** The skull bone defects in visual after 6 weeks of repair (n = 6). **(C–E)** The micro-CT images, bone defect area quantification, and bone volume analysis of the skulls repaired after 6 weeks (n = 6). **(F)** H&E staining, Masson staining, and OCN staining of bone defect areas after 6 weeks of repair. Graphs show the mean value ±SD. *p < 0.05, **p < 0.01, ***p < 0.001.

To evaluate the effect of the BMSCs with *Fgfr2* overexpression on the bone regeneration process *in vivo*, we used GelMA and/or BMSCs from the control group or the *Fgfr2* overexpression group as appropriate to treat bone defects on mouse skulls (Figure 7A). By visual observation and micro-CT scan, the *Fgfr2* overexpression BMSCs carried by GelMA greatly enhanced both the osteogenesis and regeneration in the area of the cranial defect (Figures 7B,C), and single GelMA or control BMSCs carried by GelMA also had therapeutic effects to a certain extent. Compared to the blank group, the defect area (Figure 7D) in the GelMA group decreased by 22.24% (*p* = 0.006), the control BMSCs carried by GelMA decreased it by 44.91% (*p* = 0.000), and the *Fgfr2* overexpression BMSCs carried by GelMA decreased it by 79.99% (*p* = 0.000). Consistent with the above, the BV/TV showed an upward trend as well. Compared to the blank group, the BV/TV in the GelMA group increased by 24.07% (*p* = 0.010), the control BMSCs carried by GelMA increased it by 88.65% (*p* = 0.000), and the *Fgfr2* overexpression BMSCs carried by GelMA increased it by 140.41% (*p* = 0.000).

Histological staining further demonstrated the great repair abilities of the *Fgfr2*-overexpressed BMSCs combined with GelMA on cranial bone defects (Figure 7F). Using H&E staining, obvious new bone formations could be observed in the *Fgfr2* overexpression group. The defect areas were entirely filled with fibrous soft tissues as well as new bone. Furthermore, under Masson’s trichrome staining, collagen fibers that were relatively loose were colored blue, and new generated bone was colored red. With the treatment of the control BMSCs carried by GelMA, obvious collagen fibers also appeared in the defect area. However, compared to the control BMSCs, more new bone formation could be observed after the treatment of *Fgfr2* overexpression BMSCs carried by GelMA, indicating the promotion of osteogenesis after *Fgfr2* overexpression. Corresponding to the above, the *Fgfr2* overexpression group had the highest OCN expression in the defect area as well. These results suggest that *Fgfr2* overexpression in BMSCs fulfilled significant osteogenic and potential chondrogenic ossification functions in bone regeneration. Compared to the blank group, the GelMA group and the control BMSCs group, the *Fgfr2*-overexpressed BMSC material significantly advanced ossification and regeneration in bone defect areas. In conclusion, we find that *Fgfr2* can be used as an effective gene editing target for the treatment of critical cranial bone defects in mice.

## 4 Discussion

The healing of bone defects has always been a difficult clinical challenge, especially for critical-sized bone defects caused by trauma or tumor resection that have adverse effects on patients’ prognosis and quality of life (Wei et al., 2020). For large-area bone defects, the current clinical treatments are autologous or allograft bone transplantation, but both have risks. Therefore, the use of gene-edited autologous stem cells combined with biological materials for artificial transplantation has become a new direction for possible treatment. BMSCs, which are easy to obtain and have the capacity for self-renewable and multi-directional differentiation, have already been used in the treatment of various diseases (Zhu et al., 2023a). Light-induced GelMA, as a new material with good biocompatibility, degradability, mechanical properties, and operability, has also been widely used in the repair of bone defects (Liu et al., 2016). In this paper, we found that *Fgfr2* overexpression significantly enhanced the proliferation and osteogenic differentiation potential of BMSCs and that GelMA-embedded *Fgfr2*-overexpressed BMSCs dramatically promoted the repair of critical cranial bone defects in mice. These findings demonstrated that *Fgfr2* is an essential positive regulator that mediates both endomembranous osteogenesis and endochondral osteogenesis and provide a new therapeutic method for bone regeneration.

Fibroblast growth factor receptor 2 (*Fgfr2*) is considered to be a transmembrane protein and consists of three immunoglobulin-like domains, a single hydrophobic transmembrane segment, and an intracellular tyrosine kinase domain (Dai et al., 2019). It has complex functions and is involved in many important processes within the body, such as cell proliferation and division, cell maturation and differentiation, and embryonic development (Xie et al., 2020). In the skeletal system, *Fgfr2* is an important regulator of osteogenesis and plays a role in both entochondrostosis and intramembrane osteogenesis (Sargar et al., 2017). During embryonic bone development, its expression occurs in mesenchymal stem cells and osteoblasts in bone, but not in mature osteocytes and osteoclasts (Chikazu et al., 2001; Ornitz and Marie, 2015; Mckenzie et al., 2018). As for bone fracture repair, *Fgfr2* was found to be expressed in periosteum and chondrocytes (Rundle et al., 2002), slightly upregulated during the first 4 days of fracture repair, and significantly upregulated between 9 and 14 days (Schmid et al., 2009), indicating its specific function in maintaining bone homeostasis.

*Fgfr2* has two subtypes, IIIb and IIIc, of which IIIb is mainly present in the skin and internal organs, and IIIc is widely distributed in the skeletal mesenchyme. The signaling pathways related to FGFs/FGFR2 in skeletogenesis have been explored extensively. After receiving FGF signal stimulation, tyrosine residues in the intracellular structure of FGFR2 are phosphorylated, and target proteins (e.g. PLCγ, STAT1, STAT3, and STAT5) are recruited to the cytoplasmic tail. Further downstream signaling pathways (e.g. Wnt, MAPK, AKT, ERK1/2, and STAT) are then activated by phosphorylation of those proteins, thus regulating cell growth and differentiation (Yin et al., 2008; Pfaff et al., 2016; Xie et al., 2020). Moreover, FGFR2 proteins can also enter the nucleus to promote RNA polymerase I-mediated transcription, influencing other physiological activities by regulating gene transcription (Sheng et al., 2005; Neben et al., 2014; Tuzon et al., 2019). In addition, the intracellular stability of FGFR2 is maintained by OTUB1, which inhibits FGFR2 ubiquitination to avoid its fate of lysosome degradation. Osteoblast conditional *Otub1* knockout mice showed a bone loss, suggesting an important role of *Fgfr2* in maintaining bone homeostasis (Zhu et al., 2023b).

Multiple studies have focused on abnormal *Fgfr2* mutations that are closely related to human craniosuture syndrome and bent bone dysplasia of long bones (Sargar et al., 2017), and several animal models have been established to explore the function of *Fgfr2* in bone development (e.g. *Fgfr2* ^+/S252W^; *Fgfr2* ^+/P253R^; *Fgfr2* ^+/S252W^; *Fgfr2* ^+/Y394C^; *Fgfr2IIIc* ^+/C342Y^, and *Fgfr2IIIc* ^-/-^) (Eswarakumar et al., 2002; Percival et al., 2012; Motch Perrine et al., 2019; Hoshino et al., 2023). For example, *Fgfr2* ^+/P253R^ mutation has been reported to cause impaired chondrogenesis and delayed closure of sutures, increased apoptosis of osteoblasts, and dysregulation of osteogenic differentiation; *Fgfr2IIIc* ^-/-^ mice have shown delayed ossification, reduced length of the limb bones, and reduced cell proliferation (Eswarakumar et al., 2002). In addition to its direct effect on osteogenesis, *Fgfr2* mutations can also influence osteoclasts by modulating RANKL secretion levels from osteoblasts, resulting in abnormal osteoclast activation (Xu et al., 2017; Shin et al., 2022). Consequently, *Fgfr2* has been identified as an important regulator of bone homeostasis.

However, the function of *Fgfr2* itself during the process of osteogenesis has not been fully clarified, and whether *Fgfr2* can become a gene editing target to promote bone defect repair has been unclear. Therefore, in this study, we used Lenti-virus to overexpress *Fgfr2* in BMSCs and investigated the functional outcomes. We found that *Fgfr2* promoted the proliferation, vertical and horizontal migration, anti-apoptosis ability and surface marker changing of BMSCs, indicating that *Fgfr2* may be an intrinsic factor that is required for the maintenance of stemness in BMSCs. With the addition of osteogenic induction, *Fgfr2* overexpression also promoted osteogenic differentiation and mineralization, suggesting the positive regulation of *Fgfr2* in intramembranous ossification. *Fgfr2* also plays a role in endochondral ossification. With the chondrogenic induction, BMSCs with *Fgfr2* overexpression had higher chondrogenic markers and chondrgoenic pellet formation.

As a cell membrane surface receptor, *Fgfr2* is an affinity for several *Fgf* factors (including *Fgf*2, *Fgf*9, *Fgf*20, *etc.*), which can be either autocrine or paracrine (Xie et al., 2020). However, not all *Fgf*s positively activate its downstream signaling that promotes proliferation and osteogenesis (such as *Fgf*9) (Tang et al., 2021). In our study, only *Fgfr2* was overexpressed without the exogenous addition of *Fgf*s, so we speculated that these functions caused by *Fgfr2* overexpression might be related to *Fgf* secreted by BMSCs themselves. For example, *Fgf*2 has been reported to be expressed in BMSCs and is highly expressed during bone fracture reparation (Schmid et al., 2009), and exogenous addition of *Fgf*2 can also enhance proliferation, migration and osteogenesis consistent with our experimental results (Martin et al., 1997; Charoenlarp et al., 2017; Chen et al., 2021).

Finally, we used GelMA-embedded *Fgfr2*-overexpressed BMSCs to treat critical cranial bone defects in mice and found that overexpression of *Fgfr2* significantly advanced the ossification process in the bone defect areas, showing a greater effect than traditional stem cell therapy. In all, our study clarifies the function of *Fgfr2* in BMSCs and proposes a new engineering site for the treatment of bone defects.

## Data Availability

The datasets presented in this study can be found in online repositories. The names of the repository/repositories and accession number(s) can be found below: Jianguoyun: https://www.jianguoyun.com/p/DX4CKLAQr8vJCxil4IMFIAA.
