# Supplementary material for: Overexpression of fibroblast growth factor receptor 2 in bone marrow mesenchymal stem cells enhances osteogenesis and promotes critical cranial bone defect regeneration
Source: Front Cell Dev Biol. 2023 May 17;11:1208239. doi: 10.3389/fcell.2023.1208239 (PMC10229770; doi:10.3389/fcell.2023.1208239)
Supplement: Supplementary file 1 [file Table1.docx]

Supplementary Material

Overexpression of fibroblast growth factor receptor 2 in bone marrow stem cells enhanced osteogenesis and promoted critical cranial bone defect regeneration

**Yiwen Zhou^12^†, Peixiang Zhu^12^†, Siyu Shen^2^, Yanyi Wang^12^, Baochao Li^12^, Baosheng Guo^2*^, Huang Li^12*^**

*** Correspondence:** Corresponding Author: borisguo@nju.edu.cn; lihuang76@nju.edu.cn

# Supplementary Tables

| Gene Name | Forward | Reverse |
| --- | --- | --- |
| *Gapdh* | GGCTGTATTCCCCTCCATCG | CCAGTTGGTAACAATGCCATGT |
| *Fgfr2* | CCTCGATGTCGTTGAACGGTC | CAGCATCCATCTCCGTCACA |
| *Runx2* | GACTGTGGTTACCGTCATGGC | ACTTGGTTTTTCATAACAGCGGA |
| *Alp* | CATGCCCAGTGCCTTCTGATT | TGAGATTCGTCCCTCCGCTGG |
| *Bmp4* | TGGACACCTCATCACACGAC | GCCCAATCTCCACTCCCTTG |
| *Col1a1* | GCATGAGCCGAAGCTAACCC | GTGGCAGATACAGATCAAGCATACC |
| *Col2a1* | TGAAGACACCAAGGACTGCC | GCAGTGGCGAGGTCAGTAG |
| *Col10a1* | GCTTCAGGGAGTGCCATCATC | CTCACATTGGAGCCACTAGGAATC |
| *Aggrecan* | CCCAAACAGCAGAAACAGC | GGTGGCTCCATTCAGACAA |

**Supplementary Table 1.** The primer sequence of qPCR in this experiment.
